# A randomized phase II trial of nab‐paclitaxel and gemcitabine with tarextumab or placebo in patients with untreated metastatic pancreatic cancer

**DOI:** 10.1002/cam4.2425

**Published:** 2019-07-26

**Authors:** Zishuo Ian Hu, Johanna C. Bendell, Andrea Bullock, Noelle K. LoConte, Hassan Hatoum, Paul Ritch, Hugo Hool, Joseph W. Leach, James Sanchez, Davendra P. S. Sohal, John Strickler, Ravindranath Patel, Andrea Wang‐Gillam, Irfan Firdaus, Kenneth H. Yu, Ann M. Kapoun, Eric Holmgren, Lei Zhou, Jakob Dupont, Vincent Picozzi, Vaibhav Sahai, Eileen M. O'Reilly

**Affiliations:** ^1^ Memorial Sloan Kettering Cancer Center New York New York; ^2^ Sarah Cannon Research Institute/Tennessee Oncology Nashville Tennessee; ^3^ Beth Israel Deaconess Medical Center Boston Massachusetts; ^4^ University of Wisconsin Cancer Center Madison Wisconsin; ^5^ University of Oklahoma Health Sciences Center Oklahoma City Oklahoma; ^6^ Froedtert Hospital and Medical College of Wisconsin Milwaukee Wisconsin; ^7^ Torrance Memorial Physician Network Redondo Beach California; ^8^ Virginia Piper Cancer Institute Minneapolis Minnesota; ^9^ Comprehensive Cancer Centers of Nevada Henderson Nevada; ^10^ Cleveland Clinic Cleveland Ohio; ^11^ Duke University Durham North Carolina; ^12^ Comprehensive Blood and Cancer Center Bakersfield California; ^13^ Washington University School of Medicine Saint Louis Missouri; ^14^ Oncology Hematology Cancer, Inc. Cincinnati Ohio; ^15^ David M. Rubenstein Center for Pancreatic Cancer Research New York New York; ^16^ Department of Medicine Weill Cornell Medical College New York New York; ^17^ Oncomed Pharmaceuticals Inc Redwood City California; ^18^ Virginia Mason Medical Center Seattle Washington; ^19^ University of Michigan Ann Arbor Michigan

**Keywords:** cancer stem cell, gemcitabine, nab‐paclitaxel, Notch 2/3 receptor inhibitor, Pancreatic cancer, tarextumab

## Abstract

**Purpose:**

Notch signaling dysregulation is implicated in the development of pancreatic adenocarcinoma (PDAC). Tarextumab is a fully human IgG2 antibody that inhibits Notch2/3 receptors.

**Patients and Methods:**

Aphase 2, randomized, placebo‐controlled, multicenter trial evaluated the activity of tarextumab in combination with nab‐paclitaxel and gemcitabine in patients with metastatic PDAC. Patients were stratified based on ECOG performance score and Ca 19‐9 level and randomized 1:1 to nab‐paclitaxel, gemcitabine with either tarextumab or placebo. Based on preclinical and phase Ib results suggesting a positive correlation between *Notch3* gene expression and tarextumab anti‐tumor activity, patients were also divided into subgroups of low, intermediate, and high *Notch3* gene expression. Primary endpoint was overall survival (OS) in all and in patients with the three *Notch3* gene expression subgroups (≥25th, ≥50% and ≥75% percentiles); secondary end points included progression‐free survival (PFS), 12‐month OS, overall response rate (ORR), and safety and biomarker investigation.

**Results:**

Median OS was 6.4 months in the tarextumab group vs 7.9 months in the placebo group (HR = 1.34 [95% CI = 0.95, 1.89], *P *= .0985). No difference observed in OS in the *Notch3* gene expression subgroups. PFS in the tarextumab‐treated group (3.7 months) was significantly shorter compared with the placebo group (5.5 months) (hazard ratio was 1.43 [95% CI = 1.01, 2.01]; *P *= .04). Grade 3 diarrhea and thrombocytopenia were more common in the tarextumab group.

**Conclusions:**

The addition of tarextumab to nab‐paclitaxel and gemcitabine did not improve OS, PFS, or ORR in first‐line metastatic PDAC, and PFS was specifically statistically worse in the tarextumab‐treated patients.

**Clinical trial registry no:**

NCT01647828.

## 1 INTRODUCTION

Patients with pancreatic ductal adenocarcinoma (PDAC) face a challenging prognosis. More than half of those at diagnosis have stage IV disease for which the 5‐year overall survival is 3%.^1^ Current combination cytotoxic therapies for metastatic PDAC have shown real but modest overall survival impact.^2^, ^3^ A potential explanation for either de novo or acquired treatment resistance is the presence of cancer stem cells (CSCs), cells that possess the capacity for self‐renewal, differentiation into multiple lineages, and the ability to proliferate extensively.^4^ CSCs have been shown to be more resistant to chemotherapy and radiotherapy than remaining epithelial malignant cells, persisting after therapy to drive tumor growth.^5^, ^6^ The abnormal expression of the Notch pathway, a key regulator of PDAC CSCs, has been linked to disease progression and chemotherapy resistance in metastatic PDAC.^7^, ^8^, ^9^, ^10^, ^11^

Tarextumab (OMP‐59R5) is a fully human IgG2 antibody against the Notch2 and Notch3 receptors.^12^ Preclinical studies using pancreatic xenograft models have found that treatment with the combination of tarextumab, gemcitabine, and nab‐paclitaxel induced tumor regression, decreased CSC frequency, and delayed tumor progression compared to treatment with cytotoxic therapy alone.^12^ Tarextumab also downregulated *Rg5*, a marker of developing pericytes, and facilitated pericyte recruitment to endothelial cells. Loss of *Rg5* has been reported to reduce tumor hypoxia and to normalize vasculature.^13^ Consequently, tarextumab may enhance chemotherapy sensitivity by lowering CSC frequency and reducing tumor hypoxia. Higher *Notch3* gene expression levels in these pancreatic tumor models were also found to be associated with increased sensitivity to the combination of tarextumab and gemcitabine. Based on the preclinical data, our hypothesis was that PDAC patients with higher levels of *Notch3* gene expression in tumor cells would have an enhanced potential for therapeutic benefit from the addition of tarextumab to standard therapy.

In a phase Ib study of N = 38 patients with previously untreated metastatic PDAC, tarextumab was evaluated in combination with nab‐paclitaxel and gemcitabine. The recommended phase 2 dose (RP2D) was determined to be 15 mg/kg with standard doses of the cytotoxic agents. Diarrhea, fatigue, and anemia were the most common tarextumab‐related toxicities, and the events were mostly Grade 1 or 2. The overall response rate (CR + PR) was 29%.^14^ The median PFS and OS were 5.6 and 11.6 months, respectively. Patients with high expression of *Notch3* were noted to have a PFS of 6.6 months and OS of 14.6 months. These results were deemed to compare favorably to reported PFS of 5.5 months and OS of 8.5 months in patients treated with gemcitabine and nab‐paclitaxel.^3^

Given the encouraging preclinical data, tolerable safety profile and the favorable PFS and OS results in the phase Ib study, a randomized phase II study comparing gemcitabine, nab‐paclitaxel with either tarextumab or placebo was initiated in patients with previously untreated metastatic PDAC.

## 2 PATIENTS AND METHODS

### 2.1 Study design and participants

**Figure 1:**
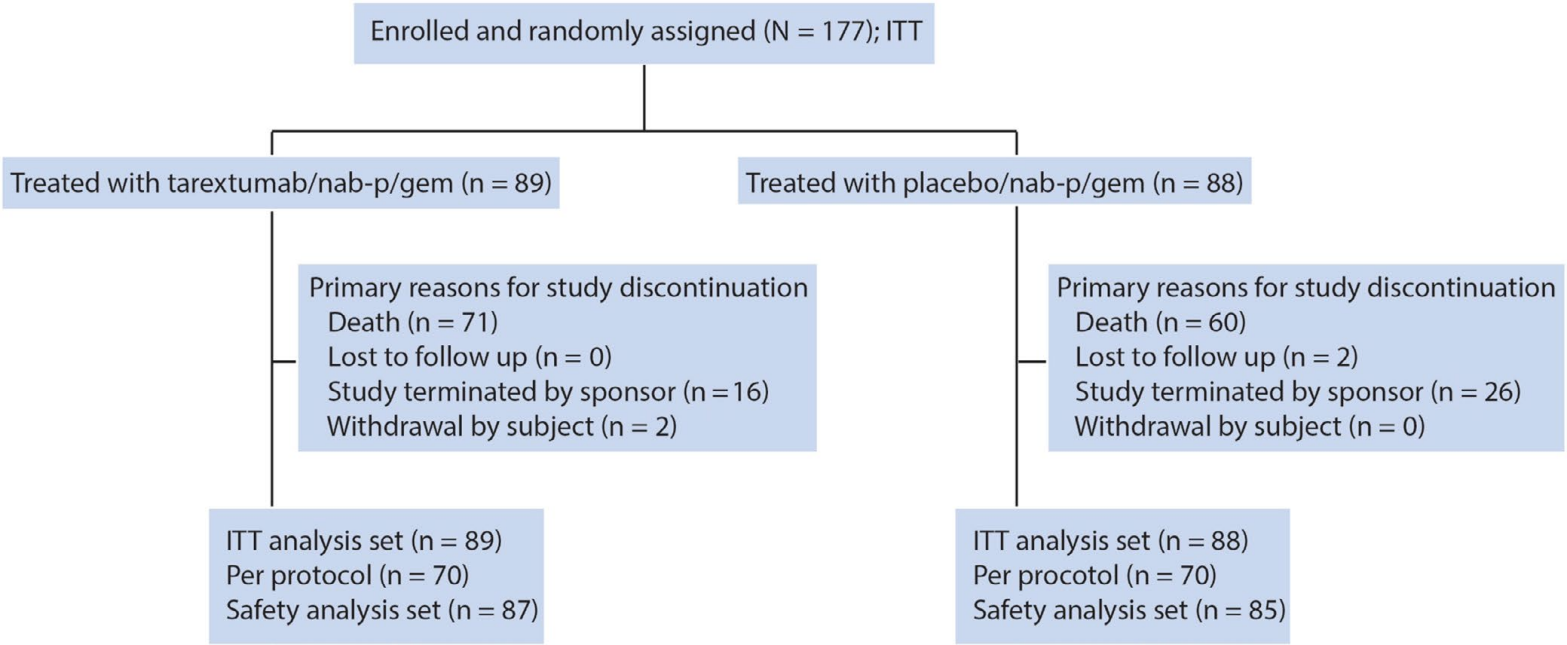
CONSORT diagram. ITT, intent to treat; nab‐p, nab‐paclitaxel; gem, gemcitabine

This was a prospective, multicenter, randomized, double‐blinded, placebo‐controlled phase II study in patients with untreated metastatic PDAC. Patients were randomized in 1:1 ratio to receive either nab‐paclitaxel, gemcitabine and placebo or nab‐paclitaxel, gemcitabine and tarextumab (Figure 1). Patients were divided into subsets based on *Notch3* gene expression levels: *Notch3* ≥ 25th percentile; *Notch3* ≥ 50th percentile; *Notch3* ≥ 75th percentile and all patients irrespective of *Notch3* expression levels.

The primary endpoint of the study was overall survival (OS). Secondary endpoints included progression‐free survival (PFS), overall response rate (ORR), duration of response (DOR), and CA19‐9 response.

Exploratory endpoints not reported herein included expression levels of epidermal growth factor receptor (EGFR), placental growth factor (PLGF), epithelial neutrophil‐activating peptide (ENA 78), and other Notch‐related genes in the serum obtained at baseline and disease progression. Circulating tumor cells (CTCs), microRNAs, and circulating endothelial cells were also evaluated and will be reported separately.

Tumor assessments were assessed by RECIST version 1.1 every 8 weeks using computed tomography or magnetic resonance imaging. Adverse events were graded using the National Cancer Institute Common Terminology Criteria for Adverse Events (CTCAE), version 4.02.^15^

Individuals (age > 18 years) with newly diagnosed, pathologically confirmed stage IV PDAC were enrolled. Eligibility criteria also included the presence of measurable disease according to RECIST version 1.1 and Eastern Cooperative Oncology Group (ECOG) performance status of 0 or 1. In additions, patients must have had formalin‐fixed, paraffin‐embedded (FFPE) tumor tissue from metastatic sites, either archived or fresh core needle biopsied for *Notch3* analysis at study entry.

Patients were required to have adequate organ function as defined by the following factors: absolute neutrophil count ≥ 1.5 × 10^9^/L, hemoglobin ≥ 9.0 g/dL, platelets > 100 × 10^9^/L (have not received hematopoietic growth factors, transfusion of blood and blood products ≥ 1 week prior to meeting the eligibility criteria), serum creatinine ≤ 1.5 mg/dL or calculated creatinine clearance ≥ 60 mL/min using the Cockcroft and Gault formula, bilirubin ≤ 1.5 × upper limit of normal (ULN), alanine aminotransferase (ALT) ≤ 3 × ULN, aspartate aminotransferase (AST) ≤ 3 × ULN), PT/INR ≤ 1.5 × ULN, aPTT ≤ 1.5 × ULN.

Patients were excluded for the following reasons: neuroendocrine tumors of the pancreas, brain metastases, prior therapy for stage IV PDAC, known human immunodeficiency virus (HIV) infection, and major surgery <4 weeks prior to the first treatment. In addition, patients with serious or unstable concomitant systemic disorder incompatible with the study such as active infection, arterial thrombosis, and symptomatic pulmonary embolism were ineligible as were patients with any disorder that would significantly compromise protocol compliance.

### 2.2 Procedures

Nab‐paclitaxel 125 mg/m^2^ and gemcitabine 1000 mg/m^2^ were administered on Days 1, 8, and 15 of every 28‐day cycle. Tarextumab or placebo was dosed at 15 mg/kg and administered on Days 1 and 15 of every 28‐day cycle. On days when tarextumab, nab‐paclitaxel and gemcitabine were given, tarextumab was administered first, then nab‐paclitaxel followed by gemcitabine. All agents were dosed until either disease progression or limiting toxicity occurred.

### 2.3 Statistical analyses

Overall survival was the primary efficacy endpoint of this study and was defined as the time from randomization until death. The hazard in the control arm was 0.09 (median 8 months) and hazard in the tarextumab arm 0.07 (median 10 months). No treatment cross‐over was permitted in the study. The Kaplan‐Meier method was used to estimate both the survival curves and the median survival time. The 95% confidence intervals for median survival times and *P*‐value for treatment effect were generated using a stratified Cox proportional hazards model.

Final analysis of the study was planned to take place at the point when 104 progression events had occurred or 10 months after the completion of enrollment, whichever occurred first. This would ensure the study had 75% power to detect a hazard ratio of 0.67 (improvement in median PFS from 5.5 months to 8.2 months) and 80% power to detect a hazard ratio of 0.65 (improvement in median PFS from 5.5 to 8.5 months) in the intention‐to‐treat (ITT) population with an associated total one‐sided type 1 error of 0.10. The data cutoff for the final analysis of survival was 6 months after the data cutoff for the final analysis of PFS or when 104 deaths have been observed, whichever occurred first.

Data were combined from all participating study sites for the analyses. All statistical testing was two‐sided and was performed at the 0.05 significance level. For continuous variables, descriptive statistics included the mean, SD, median, minimum, maximum, and the number of non‐missing values. For categorical variables, descriptive statistics included counts and percentages per category.

*Notch3* gene expression analyses were conducted as follows; *Notch3* gene expression correlated with efficacy in preclincial models. Ten primary patient derived pancreas cancer tumor xenografts were tested for efficacy in response to gemcitabine and tarextumab. Significant correlation was found between the levels of tumor‐derived *Notch3* and the efficacy of tarextumab. Responder tumors had higher levels of *Notch3* compared to nonresponders when treated with the combination of chemotherapy and the antibody. Subsequently data from the phase Ib clinical trial evaluated 32 patient samples from 40 patients enrolled in 6 dose levels of tarextumab (ranging from 2.5 to 15 mg/kg) with an 80% success rate for *Notch3* gene expression adjudication. A trend was observed for the higher levels of *Notch3* and increased time to tumor progression. Based on these collective preclinical and clinical data *Notch3* gene expression subgroups (≥25th, ≥50% and ≥75% percentiles) were chosen to best differentiate outcome for the randomized phase II trial.

### 2.4 Study oversight

The study was conducted in accordance with the Guideline for Good Clinical Practice, the ethical principles of the Declaration of Helsinki, and was approved by the institutional review board at every site. All patients provided written informed consent before enrollment.

## 3 RESULTS

### 3.1 Patients

A pre‐planned interim efficacy and safety analysis was conducted by an independent Data and Safety Monitoring Board (DSMB) on data from N = 172 patients treated by January 2016 data cutoff date. From a safety standpoint, the DSMB identified no unexpected safety signals but noted worse RR and PFS in the tarextumab treatment group and a strong trend toward lack of OS benefit in the tarextumab treatment group, irrespective of *Notch3* expression levels. The study was subsequently terminated in March 2016 by the sponsor due to futility.

One hundred and seventy‐seven patients were enrolled in the study in 25 centers throughout the United States from July 2014 to March 2016 (Table 1). Fifty‐nine per cent were male, the median age was 66 years (range 34‐88) and the majority of patients were white. Eighty‐nine patients were assigned to the tarextumab arm and N = 88 to the placebo arm.

**Table 1:** Patient baseline characteristics

	Tarextumab (n = 89)	Placebo (n = 88)	Total (n = 177)
Median age, years (range)	66 (34‐88)	66 (40‐82)	66 (34‐88)
Sex, n (%)			
Male	50 (56%)	54 (61%)	104 (59%)
Female	39 (44%)	34 (39%)	73 (41%)
ECOG score, n (%)			
0	34 (38%)	34 (39%)	68 (38%)
1	55 (62%)	54 (61%)	109 (62%)
CA 19‐9 Levels			
0 to ULN	19 (21%)	18 (20%)	37 (21%)
>ULN to 59xULN	24 (27%)	26 (30%)	50 (28%)
≥59xULN	46 (52%)	44 (50%)	90 (51%)
Primary pancreatic tumor location, n (%)^a^			
Head	39 (44%)	37 (42%)	76 (43%)
Body	34 (38%)	32 (36%)	66 (37%)
Tail	29 (33%)	32 (36%)	61 (35%)
Other	10 (11%)	9 (10%)	19 (11%)
Current site(s) of metastasis, n (%)^a^			
Pancreas	86 (97%)	83 (94%)	169 (96%)
Liver	76 (85%)	78 (89%)	154 (87%)
Lungs	39 (44%)	31 (35%)	70 (40%)
Lymph nodes	34 (38%)	45 (51%)	79 (45%)
Other	23 (26%)	24 (27%)	47 (27%)
Kidney	3 (3%)	3 (3%)	6 (3%)
Bone	5 (6%)	5 (6%)	10 (6%)
Number of metastatic sites, n (%)			
1	1 (1%)	1 (1%)	2 (1%)
2	28 (32%)	27 (31%)	55 (31%)
≥3	60 (67%)	59 (67%)	119 (67%)
Prior surgery, n (%)			
Yes	6 (7%)	6 (7%)	12 (7%)
No	83 (93%)	82 (93%)	165 (93%)
Prior radiotherapy, n (%)			
Yes	2 (2%)	1 (1%)	3 (2%)
No	87 (98%)	87 (99%)	174 (98%)
Prior systemic therapy, n (%)^b^			
Yes	1 (1%)	2 (2%)	3 (2%)
No	88 (99%)	86 (98%)	174 (98%)

^a^ Patients may be included in more than one site of disease. Percentage may add up to more than 100%.

^b^ These patients received prior systemic therapy as adjuvant therapy.

The median duration of treatment was 2.6 months (range: 0, 17.3) and 4.2 months (range: 0, 15.9) for patients in the tarextumab treatment group and the placebo group, respectively. The patients treated with tarextumab received a median of 6.0 doses (range: 1, 37). The patients in the placebo group received a median of 9.0 doses (range: 1, 35). Two placebo patients were lost to follow‐up and two tarextumab‐treated patients withdrew consent.

### 3.2 Efficacy

**Figure 2:**
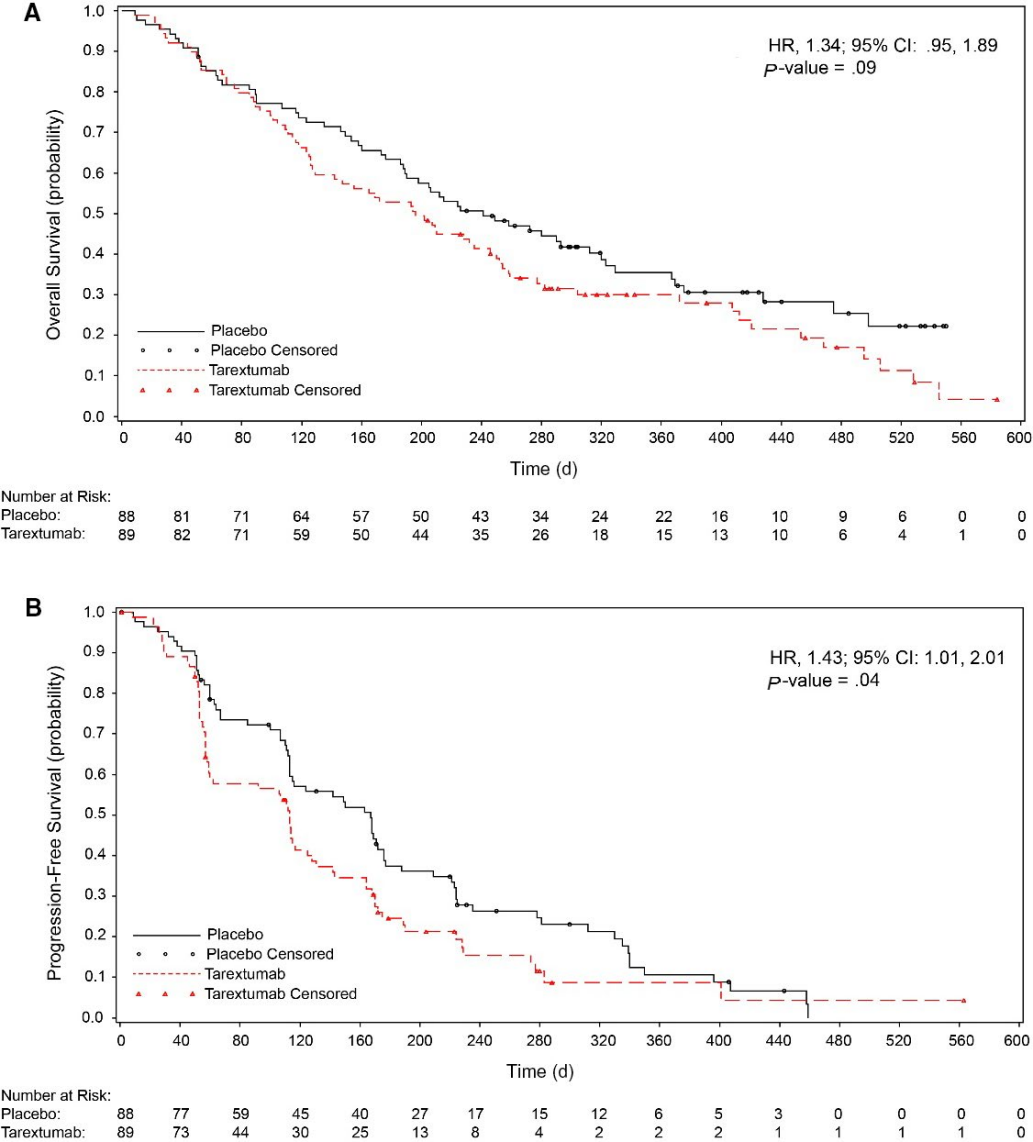
(A) Overall survival and (B) Progression‐free survival in the intent‐to‐treat population. Red, tarextumab with gemcitabine and nab‐paclitaxel; black, placebo with gemcitabine and nab‐paclitaxel

**Figure 3:**
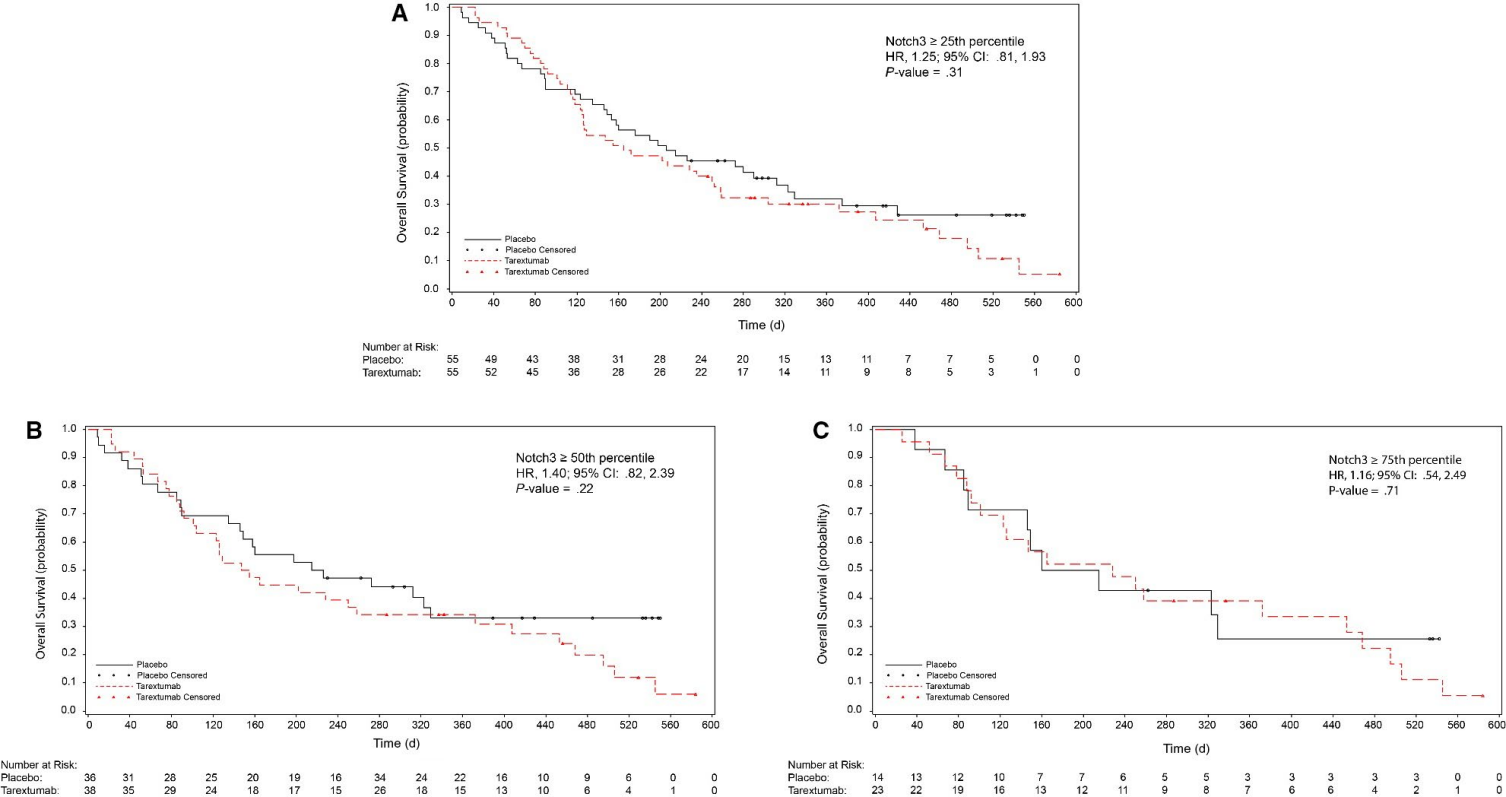
Kaplan‐Meier analysis of overall survival in the intent‐to‐treat population divided by percentile. (A) OS for patients with ≥25th percentile Notch3 expression, (B) OS for patients with ≥50th percentile Notch3 expression, (C) OS for patients with ≥75th percentile Notch3 expression. Red, tarextumab with gemcitabine and nab‐paclitaxel; black, placebo with gemcitabine and nab‐paclitaxel

The median OS was 6.4 months (95% CI = 4.17, 8.2) in the tarextumab group compared with 7.9 months (95% CI = 6.18, 10.52) in the placebo group (Figure 2A). No statistically significant difference was observed in OS in the tarextumab treatment group compared with the placebo group (HR = 1.34 [95% CI = 0.95, 1.89], *P* = .09). No statistically significant difference was observed in OS in patients with *Notch3* ≥ 25th percentile, *Notch3* ≥ 50th percentile, or *Notch3* ≥ 75th percentile (Figure 3).

The median PFS was 3.7 months (95% CI = 1.94, 4.21) in the tarextumab group and 5.5 months (95% CI = 3.72, 5.79) for the placebo group (Figure 2B**)**. The PFS in the tarextumab treatment group was significantly shorter compared with the placebo group (HR 1.43 [95% CI = 1.01, 2.01]; *P* = .04). No statistically significant difference was observed in PFS in patients with *Notch3* ≥ 25th percentile, *Notch3* ≥ 50th percentile, or *Notch3* ≥ 75th percentile.

There was no significant difference in ORR (*P* = .087) between the placebo‐treated group (31.8%; 95% CI = 22.3%, 42.6%) compared with the tarextumab‐treated group (20.2%; 95% CI = 12.4%, 30.1%) (Table 2). There was no significant difference in ORR between the Notch3 subgroups and placebo treatment groups.

**Table 2:** Treatment response

Response^a^	Tarextumab + nab‐paclitaxel + gemcitabine (n = 89)	Placebo + nab‐paclitaxel + gemcitabine (n = 88)
Overall response rate	18 (20%)	28 (32%)
Partial response	18 (20%)	28 (32%)
Stable disease	31 (35%)	36 (41%)
Progression of disease (POD)	21 (24%)	6 (7%)
Not Evaluable	—	1 (1%)
Clinical POD (no follow up imaging)	19 (21%)	17 (19%)

^a^ Per RECIST v1.1

The median duration of response (DOR) was 3.7 months (95% CI = 1.64, 5.52) in the tarextumab group and 3.6 months (95% CI = 2.14, 5.59) in the placebo group. No statistically significant difference was observed in the DOR between the two groups (*P* = .99).

CA19‐9 response was defined as a decrease of 50% or more in baseline CA19‐9 at any time post‐baseline. There was no statistically significant difference (*P* = .15) in response between the placebo group (47.7%) and the tarextumab‐treated group (37.1%).

### 3.3 Safety

Treatment‐emergent adverse events (TEAEs) are summarized in Table 3. TEAEs were reported in 93% of the tarextumab group and 80% of the placebo group. There were no statistically significant differences in the rate of adverse events between the groups. Diarrhea (72% vs 40%), nausea (41% vs 31%), and thrombocytopenia (49% vs 25%) were noted to occur more frequently in the tarextumab treatment group.

**Table 3:** Treatment‐emergent adverse events related to tarextumab with incidence of at least 10% by system organ class

System organ class	Placebo (n = 85)	Tarextumab (n = 87)
Gastrointestinal disorders		
Diarrhea	34 (40%)	63 (72%)
Nausea	26 (31%)	36 (41%)
Vomiting	14 (16%)	19 (22%)
General disorders		
Fatigue	50 (59%)	45 (52%)
Fever	10 (12%)	8 (9%)
Hematologic disorders		
Thrombocytopenia	21 (25%)	43 (49%)
Anemia	22 (26%)	25 (29%)
Neutropenia	15 (18%)	8 (9%)
Metabolism and nutrition disorders		
Decreased appetite	11 (13%)	15 (17%)
Dehydration	10 (12%)	8 (9%)
Nervous system disorders		
Dysgeusia	8 (9%)	11 (13%)
Respiratory, thoracic and mediastinal disorders		
Epistaxis	1 (1%)	9 (10%)
Patients with Grade 3 or 4 TEAEs		
Diarrhea	2 (2%)	15 (17%)
Nausea	1 (1%)	6 (7%)
Fatigue	9 (11%)	13 (15%)
Thrombocytopenia	6 (7%)	19 (22%)
Anemia	8 (9%)	14 (16%)
Neutropenia	10 (12%)	3 (3%)

Fifty‐one (60%) patients in the placebo and N = 58 (66.7%) patients in the tarextumab group reported one or more serious AEs (SAE). In the tarextumab group, 22 (25.3%) patients had Grade 3 and 3 (3.4%) patients had Grade 4 SAE. Fourteen (16.1%) SAEs in the tarextumab‐treated group and 16 (18.8%) in the placebo‐treated group resulted in patient death; none of these SAEs were considered related to study drug treatment.

TEAEs in 8 tarextumab‐treated patients resulted in treatment interruptions and 6 patients experienced tarextumab dose reductions due to TEAEs. TEAEs led to treatment discontinuation in 7 patients. A total of 126 (73.3%) patients died within 30 days of study discontinuation, 69 (79.3%) tarextumab‐treated patients and 57 (67.1%) placebo‐treated patients. None of the deaths were adjudicated as related to tarextumab.

## 4 DISCUSSION

Strong preclinical evidence with patient‐derived xenograft (PDX) pancreatic models and phase Ib trial data provided a compelling rationale for the clinical evaluation of Notch signaling inhibition combined with gemcitabine and nab‐paclitaxel therapy in a randomized phase 2 trial.^12^ Tarextumab treatment in combination with gemcitabine in PDX models resulted in decreased *Notch2* and *Notch3* expression, increased apoptotic cell death, decreased tumor cell density, and increased vessel perfusion and decreased hypoxia intratumorally.^12^ Mean gene expression levels of *Notch3* were noted to be significantly higher in responders compared to nonresponders, suggesting that higher *Notch3* expression may be a useful biomarker of sensitivity to tarextumab treatment.

In view of the encouraging preclinical findings, early phase trials were initiated in metastatic PDAC and extensive stage small cell lung cancer. Results from both phase Ib clinical trials were potentially promising, with reported response rates of 84% and 74% in the small cell lung and pancreatic cancer trials, respectively.^14^, ^16^ Analysis of the phase Ib clinical trial in PDAC demonstrated a PFS of 5.6 months and OS of 11.6 months in patients treated with tarextumab in combination with gemcitabine and nab‐paclitaxel. In the subgroup of patients with high *Notch3* expression, PFS and OS were 6.6 and 14.6 months, respectively.

However, both the small cell lung cancer and PDAC randomized phase II clinical trials failed to meet their primary objective of a statistically significant improvement in OS. In the trial reported herein in PDAC, the PFS for the tarextumab treatment group was shorter compared to the placebo group. Analysis of the Notch3 subgroups also found no difference in PFS and OS between the subgroups. To summarize, we found no benefit in adding tarextumab to standard cytotoxic therapy in this randomized phase II trial of patients with metastatic PDAC, with a statistically significant decrease in median PFS and concerning negative trend in OS for some pre‐specified subgroups. It is possible that some of the worse outcome was attributed to higher toxicity in the tarextumab vs placebo‐treated patients resulting in a lower median treatment duration (2.6 vs 4 months) and lower median number of treatment doses (6 vs 9 doses).

The discordances between the preclinical along with the early clinical development (phase Ib trial) results and the randomized phase II results are concerning and may be attributed to differences in xenograft development, the patient population in the clinical trials, as well as the pleotropic nature of the Notch receptors and unrecognized contributions from the PDAC stroma. The human xenograft tumors used for the preclinical heterotopic implant model were obtained from stage III PDAC patients without metastases whereas the clinical trial treated metastatic PDAC patients. Compared to heterotopic models, orthotopic PDAC models, where tumor cells are implanted directly into the pancreas, retain a greater proportion of stromal components and develop locoregional and distant metastases.^17^, ^18^ Consequently, the preclinical results may have been more predictive of outcome for a localized PDAC population. In the phase Ib clinical trial, 11/38 patients (27.5%) patients had ≥3 metastatic sites and 14 (35.0%) patients had two metastatic sites. In contrast, 67.2% of patients in the phase II clinical trial had ≥3 metastatic sites and 31.1% had two metastatic sites. Given the differences between the heterotopic PDX model and patient population, it is difficult to ascertain what roles Notch2 and Notch3 inhibition may play in promoting or inhibiting disease progression in a locoregional vs metastatic setting. An orthotopic or genetically engineered mouse models may promote better understanding in the future.

*Notch1* inhibition has also been reported to cause an increase in liver metastases from neuroblastoma and breast cancer cells and may support early angiogenesis and growth of micrometastases within the liver.^19^ The preclinical studies noted that tarextumab treatment alone and not combination therapy, decreased Notch1 intracellular domain levels in the xenograft OMP‐PN17 PDAC tumor.

This randomized phase II clinical trial also found no significant difference in PFS and OS between the low, intermediate, and high *Notch3* subgroups. Further analyses of the exploratory biomarkers, including Notch‐related genes, CTCs, and circulating endothelial cells collected in this trial are ongoing and may be informative.

The exact role of Notch signaling in PDAC remains an area of significant debate, with some evidence supportive of an oncogenic role while other results have been more suggestive of its function as a tumor suppressor. Notch receptors and ligands have been found to be overexpressed in human and mouse PDAC cells, and have been implicated in the progression of pancreatic intraepithelial neoplasia (PanIN) lesions.^10^, ^20^, ^21^, ^22^ Reports have differed, however, on which specific Notch receptor is central in the progression of PanIN and PDAC lesions, with results supporting Notch1,^23^ Notch2,^21^ and Notch3.^24^ Alternatively, loss of Notch1 in a KRAS‐driven PDAC mouse model resulted in increased tumor incidence and progression, implicating Notch signaling as a potential tumor suppressor.^25^

The discrete, collective, and relative contributions of the individual Notch receptors to tumor development in PDAC patients remain to be resolved. Further research into individual Notch inhibitors and agonists may help guide future clinical trials involving the Notch pathway and cytotoxic chemotherapy. To additionally note, a randomized phase II trial evaluating an anti‐Delta‐like ligand 4 (DLL4) targeted agent (demcizumab), which is an inhibitor of the Notch pathway or placebo, combined with gemcitabine and nab‐paclitaxel observed no improvement in the primary endpoint of PFS compared to standard chemotherapy and similarly there was no difference in overall survival (HR 1.02).^26^ The collective data suggest that targeting the Notch pathway to date has little clinical utility in PDAC.

To sum up, the addition of tarextumab to gemcitabine and nab‐paclitaxel in untreated advanced PDAC did not improve outcomes over standard therapy, and specifically PFS was statistically worse in the tarextumab‐treated group. This trial provides significant insights into the importance of preclinical modeling in optimal and relevant model systems and underscores the need for randomized evaluation of experimental agents.

## CONFLICT OF INTEREST

J. Bendell: Research grant from OncoMed for performance of the study covered by the submitted work; payment to her institution for consulting/advisory services and/or clinical trial activities from the following for activities outside of the study covered by the submitted work: Bristol Myers Squibb, Roche, Merck, Taiho Oncology, Amgen, Genentech, Merrimack, Celgene, MedImmune, Seattle Genetics, Daiichi Sankyo, Janssen, Translational Drug Development, Five Prime Therapeutics, Moderna Therapeutics, Tolero, Evelo Biosciences, Array Therapeutics, Forma Therapeutics, Tanabe Research Laboratories, BeiGene, Continuum Clinical, Cerulean, AbbVie, AstraZeneca, EMD Serono, Ipsen Biopharma, Incyte, Novartis, Eisai, Pfizer, Millennium, Imclone, Boston Biomedical, CALGB, Acerta Pharma, Lilly, Gilead Sciences, Leap Therapeutics, Macrogenics, OncoMed Pharmaceuticals, Takeda Pharmaceuticals, Rgenix, Novocure, Merus, NV, Blueprint Medicine, Array Biopharma, ARMO Biosciences, Agios. From time to time, the companies listed have reimbursed Dr Bendell for travel expenses and/or provided meals at meetings held in connection with her services. A. Bullock: Consulting/Advisory Boards: Eisai, Exilexis, Taiho. N. LoConte: Advisory Boards: Celgene, Bayer. Consultant: Abbvie, AstraZenica. D. Sohal: Consulting: Perthera. Honoraria: Foundation Medicine. Travel/Accomodation/Expense reimbursement: Foundation Medicine. Research funding: Agios, Bayer, Bristol‐Myers Squibb, Celgene, Genentech, Incyte, Loxo, Novartis, OncoMed. J. Strickler: Consulting/Advisory: Amgen, Genentech‐Roche, Bayer, Celgene, Chugai, AstraZenica, Seattle Genetics, OncoMed. Research Funding: Amgen, Abbvie, Gilead Sciences, Roche/Genentech, Exelixis, OncoMed, Sanofi, Regeneron, MedImmune, Cascadian Therapeutics, Seattle Genetics, Macrogenics, Leap Therapeutics, Nektar. A. Wang‐Gillam: Advisory Boards: Ipsen, Merrimack, BMS, Tyme, Repugene, Jacobio, Vicus. Research funding: BMS. Biomed Valley, Pfizer, Halozyme, AstraZeneca, Bayer, Xcovery, Novartis, Lily, Aduro, Verastem. Tyme, Boston Biomedical, Newlink Genetics, Precision Biologicis. K. Yu. Consulting/Advisory: Halozyme, Ipsen. Research Funding: Halozyme, BMS. A. Kapoun: Employee OncoMed. L. Zhou: Pain consultant to OncoMed. Stock ownership: OncoMed. J. Dupont: Employee OncoMed. Stock ownership: OncoMed. E. Holmgren: Employee OncoMed. Stock ownership: OncoMed. E. O'Reilly: Research funding to MSK: Genentech, Roche, BMS, Halozyme, Celgene, MabVax Therapeutics, ActaBiologica, OncoMed, Momenta Pharmaceuticals, Parker Institute, AstraZenica, Silenseed, Incyte, Pfizer, Polaris, Lustgarten Foundation, NCI‐CTEP. Consulting/Advisory: Cytomx, BioLineRx, Targovax, Halozyme, Celgene, Bayer, Loxo, Polaris. All remaining authors have declared no conflicts of interest.

## AUTHORS' CONTRIBUTIONS

Zishuo Ian Hu: Writing–original draft and writing–review and editing, formal analysis. Johanna C. Bendell: Investigation, funding, acquisition, formal analysis and resources, project administration. Andrea Bullock: Investigation, formal analysis and resources. Noelle K. LoConte: Investigation, formal analysis and resources. Hassan Hatoum: Investigation, formal analysis and resources. Paul Ritch: Investigation, formal analysis and resources. Hugo Hool: Investigation, formal analysis and resources. Joseph W. Leach: Investigation, formal analysis and resources. James Sanchez: Investigation, formal analysis and resources. Davendra PS Sohal: Investigation, formal analysis and resources. John Strickler: Investigation, formal analysis and resources. Ravindranath Patel: Investigation, formal analysis and resources. Andrea Wang‐Gillaam: Investigation, formal analysis and resources. Irfan Firdaus: Investigation, formal analysis and resources. Kenneth H. Yu: Investigation, formal analysis and resources. Ann M. Kapoun: Conceptualization, funding acquisition, investigation, methodology, formal analysis and resources. Eric Holmgren: Conceptualization, funding acquisition, investigation, methodology, formal analysis and resources. Lei Zhou: Conceptualization, funding acquisition, investigation, methodology, formal analysis and resources. Jakob Dupont: Conceptualization, funding acquisition, investigation, methodology, formal analysis and resources. Vincent Picozzi: Conceptualization, investigation, methodology, formal analysis and resources. Vaibhav Sahai: Investigation, formal analysis and resources. Eileen M. O'Reilly: Conceptualization, funding, acquisition, investigation, methodology, project administration, supervision, writing–original, and writing–review and editing. Precis: A phase 2 study of tarextumab, an anti‐Notch 2/3 inhibitor, in combination with gemcitabine and nab‐paclitaxel in untreated advanced PDAC did not improve outcomes over standard therapy. PFS was statistically worse in the tarextumab‐treated group.
